# Effect of β-tricalcium phosphate grafting on implant stability and peri-implant radiodensity following immediate implant placement in sites with jumping gaps exceeding 2 mm: a randomized controlled trial

**DOI:** 10.1186/s12903-026-09810-5

**Published:** 2026-09-21

**Authors:** Alaa A. Salem, Mohamed S. Hammed, Ahmed M. ElRawdy, Hesham ElHawary

**Affiliations:** 1 Department of Oral and Maxillofacial Surgery, Faculty of Dentistry, Suez Canal University, Ismailia, Egypt; 2 Department of Oral and Maxillofacial Surgery, Faculty of Dentistry, Suez Canal University, Ismailia, Egypt; 3 Department of Oral and Maxillofacial Surgery, Faculty of Dentistry, Cairo University, Cairo, Egypt

**Keywords:** Immediate dental implant, β-tricalcium phosphate, Bone graft, Osseointegration, Implant stability, Radiodensity, Jumping gap

## Abstract

**Background:**

Whether grafting peri-implant jumping gaps exceeding 2 mm during immediate implant placement improves outcomes remains uncertain. This trial evaluated the effect of β-tricalcium phosphate (β-TCP) grafting on implant stability and peri-implant radiodensity in such sites.

**Methods:**

In this prospective, parallel-group randomized controlled trial, 16 patients (8 per group) receiving 39 immediate maxillary anterior/premolar implants at sites with a jumping gap exceeding 2 mm were randomized 1:1 to no grafting (control) or injectable β-TCP (test); the patient was the unit of analysis. The pre-specified primary comparison was implant stability quotient (ISQ) at 6 months. Peri-implant radiodensity was monitored on standardized periapical radiographs (grayscale value) at baseline, 3, and 6 months, and quantified by cone-beam CT (CBCT) at 6 months only (ALARA principle); CBCT values are relative, arbitrary gray-value radiodensity, not true Hounsfield units. Between-group differences were analyzed with independent-samples t tests, within-group changes with repeated-measures ANOVA, and group×time interactions with mixed-design ANOVA (α = 0.05).

**Results:**

No statistically significant between-group differences were detected in baseline age or jumping-gap width. No significant between-group difference in ISQ was detected at any time point, and the ISQ group×time interaction was not significant (*p* = 0.49); ISQ increased significantly over time in both groups. Periapical grayscale value increased significantly faster in the test group on unadjusted analysis (group×time interaction, *p* = 0.009), but this was no longer significant after adjusting for a small baseline difference (ANCOVA, *p* = 0.39). On 6-month CBCT, radiodensity was significantly higher in the test group (1224.0 ± 237.0) than control (931.9 ± 100.4 arbitrary units; mean difference 292.1; 95% CI 87.7 to 496.5; *p* = 0.010), a secondary outcome not included in the a priori sample-size calculation.

**Conclusions:**

β-TCP grafting of jumping gaps exceeding 2 mm did not significantly affect ISQ or baseline-adjusted periapical grayscale value. The test group showed significantly higher 6-month CBCT-derived radiodensity, but this was not prospectively powered and may partly reflect residual graft material, so it should be interpreted cautiously. Larger, adequately powered equivalence trials are needed before conclusions about grafting necessity can be drawn.

**Trial registration:**

ClinicalTrials.gov, NCT07709676. Registered 2026-07-12. Retrospectively registered.

**Supplementary Information:**

The online version contains supplementary material available at https://doi.org/10.1186/s12903-026-09810-5.

## Background

Dental implant success depends fundamentally on osseointegration [1, 2]. Immediate implant placement, performed at the time of extraction, offers advantages including preserved alveolar bone volume, fewer surgical interventions, and shorter treatment time [3, 4] but introduces the challenge of the “jumping gap”—the horizontal discrepancy between the implant surface and the socket wall [5]. Gaps exceeding 2 mm may compromise primary stability and increase the risk of marginal bone loss, particularly in the esthetic zone [6].

Defects beyond this threshold may not heal predictably through spontaneous blood-clot formation alone and can favor fibrous encapsulation over bone formation [7] leading many clinicians to graft the defect to support regeneration and implant stability, consistent with a systematic review and meta-analysis reporting less horizontal ridge shrinkage with a regenerative procedure at immediate implant sites and no significant difference in implant survival [8].

β-Tricalcium phosphate (β-TCP) is a biocompatible, osteoconductive, biodegradable synthetic graft material whose composition resembles natural bone [9, 10]. It supports new bone ingrowth in defects and is gradually resorbed and replaced by native bone [11]. An injectable formulation was used here for its ability to adapt to irregular peri-implant defects while maintaining particle stability during healing.

Whether the jumping gap requires grafting remains controversial, particularly beyond 2 mm. This randomized controlled trial evaluated the effect of β-TCP grafting on implant stability and peri-implant radiodensity after immediate implant placement in sites with a jumping gap greater than 2 mm, testing the null hypothesis that grafting produces no difference in these outcomes.

## Methods

### Study design and ethical approval

**Fig. 1:**
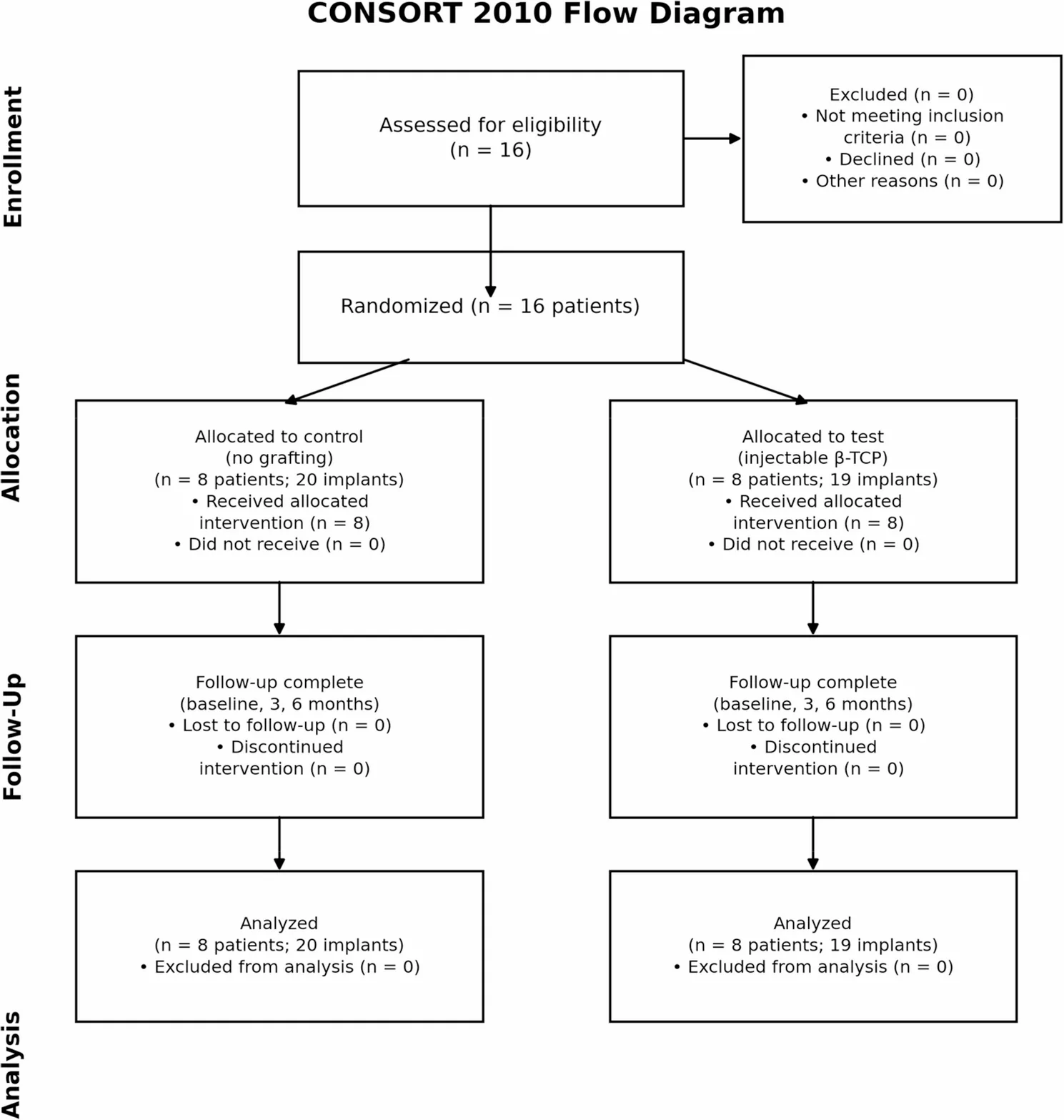
CONSORT 2010 flow diagram showing participant enrollment, allocation, follow-up, and analysis. Sixteen patients were assessed for eligibility and randomized (eight per group; no exclusions); all patients completed follow-up to 6 months with no losses and were included in the final analysis (control, 8 patients/20 implants; test, 8 patients/19 implants)

This study was conducted as a prospective, parallel-group randomized controlled trial at the Department of Oral and Maxillofacial Surgery, Faculty of Dentistry, Suez Canal University, from December 2024 to November 2025. Ethical approval was obtained from the Research Ethics Committee, Faculty of Dentistry, Suez Canal University (approval no. 682/2023), and all participants provided written informed consent in accordance with the Declaration of Helsinki. The trial was designed, conducted, and reported in accordance with the CONSORT 2010 guidelines. Participant flow through enrollment, allocation, follow-up, and analysis is presented in the CONSORT flow diagram (Fig. 1), and a completed CONSORT 2010 checklist is provided as an additional file.

### Sample-size calculation

The sample size was calculated for the primary outcome (ISQ) using the standard formula for comparing two means, *n* = 2(Z₁₋α⁄₂ + Z₁₋β)² × σ² / Δ²,10 with a two-sided significance level of α = 0.05 (Z = 1.96) and statistical power of 1 − β = 0.80 (Z = 0.84), assuming a standard deviation of 4.07 ISQ units [12] and a clinically relevant difference of 6 ISQ units, consistent with reported detectable ISQ differences of approximately 5–9 units [13, 14] (no formally established minimal clinically important difference exists for ISQ). This yielded 7.2 subjects per group, rounded up to 8 per group (16 total). No inflation for anticipated loss to follow-up was incorporated; no participants were subsequently lost to follow-up. The calculation was based on the patient, not the implant, as the pre-specified unit of analysis. Because it was based exclusively on the primary ISQ outcome, the study was not prospectively powered for the radiographic secondary outcomes, whose estimates should be interpreted with appropriate caution (see Limitations).

### Participants

Sixteen patients (eight per group) requiring extraction of non-restorable maxillary anterior and premolar roots were enrolled and received 39 immediate implants; patients with more than one qualifying site received more than one implant, with the patient (not the implant) as the unit of analysis throughout. Inclusion criteria were age 20–50 years, jumping gap > 2 mm, adequate inter-arch space, and good compliance. Exclusion criteria were systemic disease affecting healing, smoking > 10 cigarettes/day, parafunctional habits, poor oral hygiene, and active periodontal disease.

All implants shared the same tapered, triple-lead-thread, internal-conical-hex design (grade 23 titanium alloy) with an SLA acid-etched surface (Supplementary Table S1 gives diameter/length by patient). All sites were fresh extraction sockets with intact bony walls and no dehiscence or fenestration; because socket morphology was therefore uniform across sites, it was not analyzed as a covariate. Baseline jumping-gap dimensions are reported in Table 1, consistent with reporting conventions in comparable immediate-implant RCTs [15].

**Table 1:** Sample composition, allocation, and baseline characteristics

Variable	Control (*n* = 8)	Test (*n* = 8)	*p* value
Total implants, n	20	19	—
Mean implants per patient	2.5	2.4	—
Range (min–max) implants per patient	1–3	1–3	—
Female sex, n (%)	7 (87.5)	8 (100)	0.301
Male sex, n (%)	1 (12.5)	0 (0)	0.301
Age, years (mean ± SD)	46 ± 5.6	43 ± 4.8	0.269
Jumping gap, mm (mean ± SD)	2.94 ± 0.85	3.15 ± 0.94	0.372

A 1:1 allocation ratio was used. The groups included 20 and 19 implants, respectively; the distribution of implants per patient is presented descriptively

*SD* standard deviation

### Randomization and blinding

The allocation sequence was generated by an investigator not involved in surgery (M.S.H.) using a computerized randomization tool (www.randomizer.org), 1:1, with no stratification, and concealed via sequentially numbered, opaque, sealed envelopes opened only at intervention. Participants were enrolled by the operating surgeon (A.A.S.) and randomized at enrolment, before the number of implants required was determined. Outcome assessors, including the radiodensity readers, were blinded to allocation. Eligibility (jumping gap > 2 mm) was assessed preoperatively by CBCT virtual planning at the patient level, then confirmed intraoperatively with a periodontal probe immediately before allocation was revealed. No site or patient was excluded after randomization because the intraoperative measurement confirmed the preoperative estimate at every site in this cohort (CONSORT flow diagram, Fig. 1); a site failing to exceed 2 mm intraoperatively would have been excluded from site-level analyses without altering the patient’s allocation, but this did not occur.

### Preoperative assessment

**Fig. 2:**
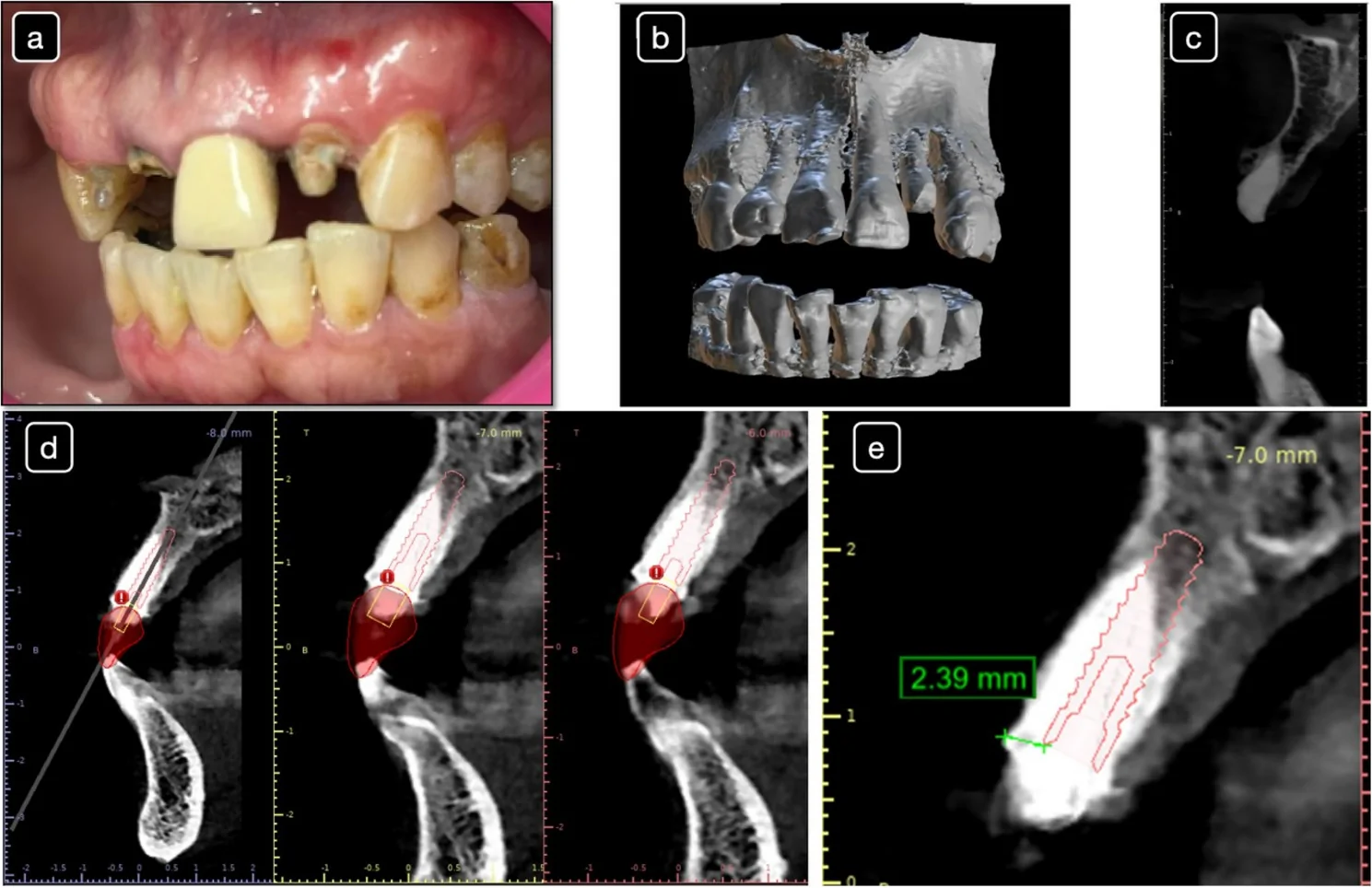
Preoperative assessment and virtual planning. **a** Preoperative clinical photograph. **b** Three-dimensional CBCT reconstruction. **c** Sagittal CBCT view of the site of interest confirming the absence of periapical pathosis. **d** Prosthetically driven virtual planning of the implant in its ideal position. **e** Virtual measurement of the jumping gap

**Fig. 3:**
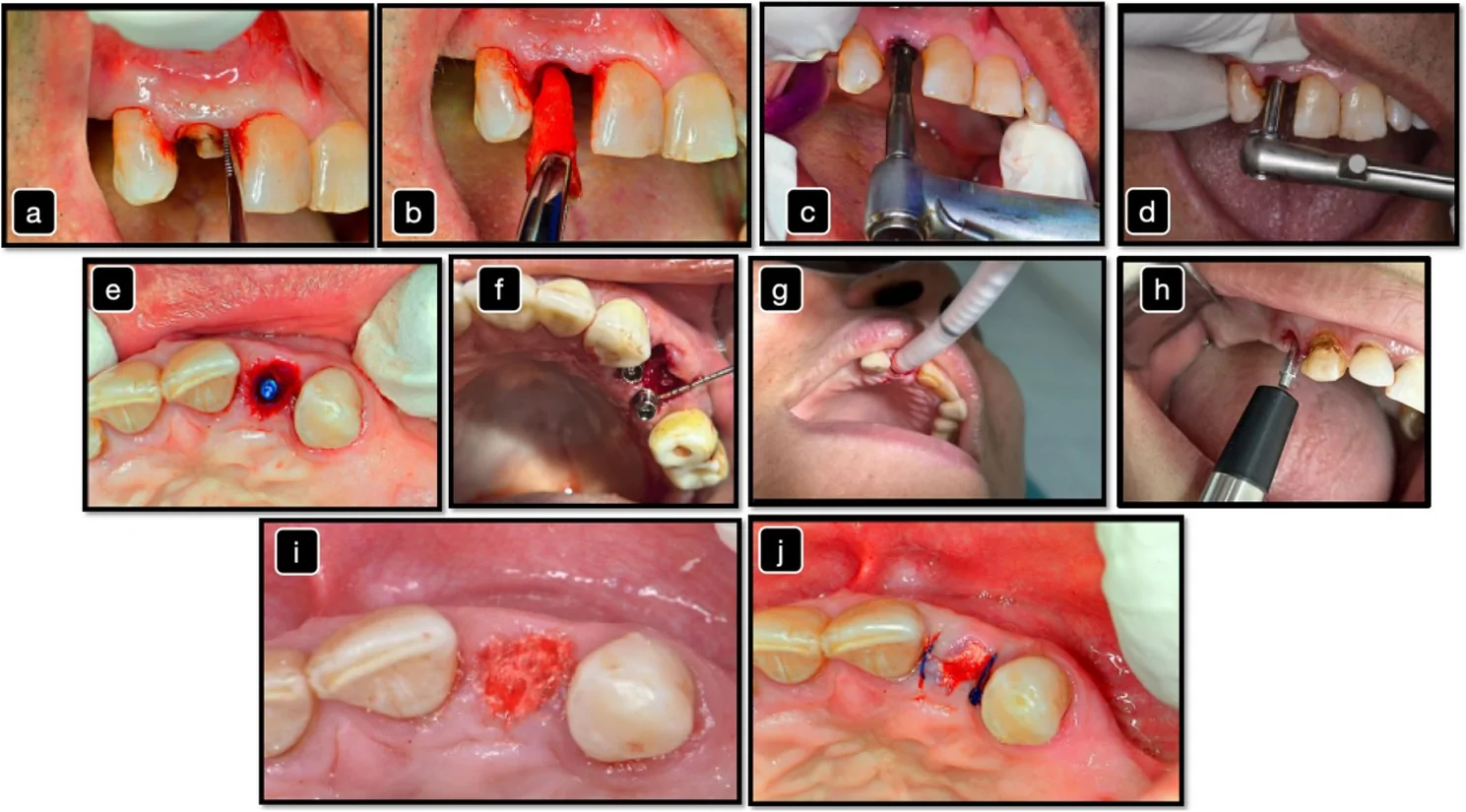
Surgical procedure. **a** Atraumatic extraction with a periotome. **b** Tooth removal with forceps. **c** Osteotomy preparation. **d** Implant placement. **e** Occlusal view of the implant platform. **f** Measurement of the jumping gap with a periodontal probe. **g** Injection of β-TCP around the implant (test group). **h** Application of the SmartPeg and ISQ measurement. **i** Placement of a gelatin sponge over the cover screw and platform. **j** Wound closure with 4 − 0 polypropylene interrupted sutures

All patients underwent clinical and radiographic evaluation, with medical and dental history recorded to exclude systemic contraindications (Fig. 2a). CBCT was performed preoperatively to assess local anatomy, plan implant position, and estimate the jumping gap on the virtual plan (Fig. 2b-e). Preoperative preparation included oral-hygiene instructions and chlorhexidine mouthwash (0.12%, three times daily for 3 days preoperatively and 2 weeks postoperatively). Antibiotic prophylaxis followed current evidence favoring a single preoperative dose over extended postoperative courses [16]: amoxicillin 2 g orally 1 h before surgery (clindamycin 600 mg for penicillin-allergic patients). The jumping gap was defined as the horizontal distance, perpendicular to the implant long axis at the alveolar crest, between the implant’s outer buccal surface and the socket’s buccal wall; it was estimated preoperatively on CBCT (Fig. 2e) and confirmed intraoperatively with a calibrated periodontal probe (Fig. 3f). Intraoperative measurements were taken once, by a single examiner, without independent replication, a limitation of the eligibility protocol (see Limitations).

### Surgical procedure

All procedures were performed under local anesthesia (4% articaine with 1:100,000 epinephrine). Atraumatic extraction was performed with a periotome and forceps to preserve alveolar bone (Fig. 3a, b), followed by socket debridement and saline irrigation. Osteotomy preparation favored a prosthetically driven, slightly palatal position to preserve the buccal plate, extending 3–4 mm beyond the socket apex to engage apical/palatal bone and enhance primary stability while permitting a controlled buccal jumping gap (Fig. 3c). Implants were placed at crestal bone level per the manufacturer’s protocol (Fig. 3d, e); the jumping gap was confirmed clinically with a periodontal probe, and only sites exceeding 2 mm were included (Fig. 3f). No grafting was performed in the control group; in the test group, the gap was filled after implant placement with an injectable β-TCP synthetic bone substitute in a ready-to-use putty carrier (Bonegraft Biomaterials Co., Ltd., Daegu, South Korea) (Fig. 3g). Primary stability was assessed by resonance frequency analysis (Osstell), expressed as ISQ (Fig. 3h). A gelatin sponge was placed (Fig. 3i) and the wound closed with interrupted 4 − 0 polypropylene sutures (Fig. 3j).

#### Postoperative care

Patients were instructed to apply cold packs during the first 24 h, avoid rinsing or spitting, follow a soft diet, and avoid mechanical trauma to the surgical site. Non-steroidal anti-inflammatory drugs were prescribed as needed for analgesia. Chlorhexidine mouthwash was used twice daily from the second postoperative day for 1 week.

### Outcome measures

**Fig. 4:**
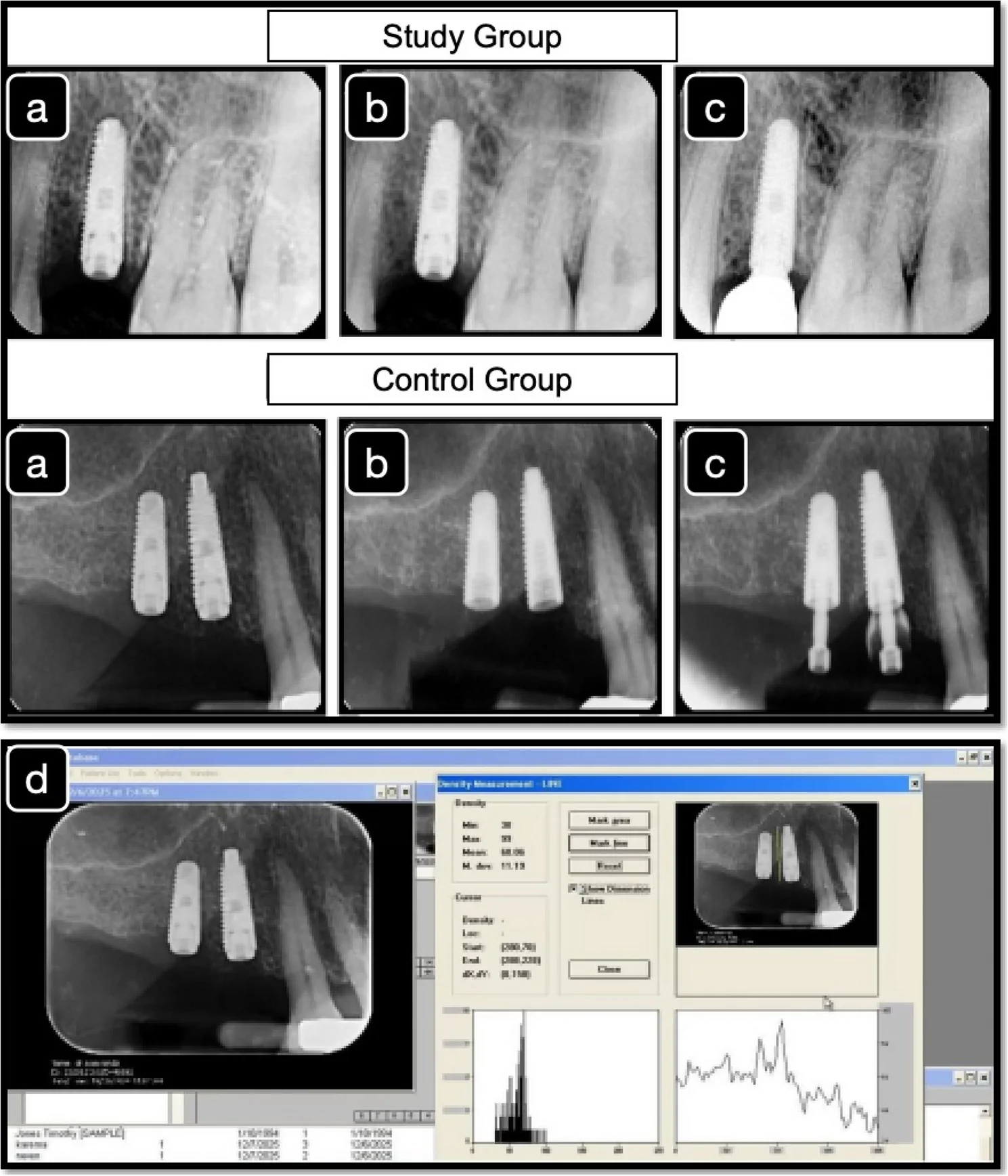
Radiographic follow-up and radiodensity assessment. Standardized periapical radiographs at the immediate postoperative (**a**), 3-month (**b**), and 6-month (**c**) intervals for the test and control groups, and (**d**) measurement of peri-implant radiodensity using Digora software

The primary outcome was implant stability (ISQ), recorded at baseline, 3 months (healing-abutment placement), and 6 months (before final restoration); the pre-specified primary comparison was the between-group difference in ISQ at 6 months, fixed at the design stage before results were examined. The secondary outcome was peri-implant radiodensity, assessed by two complementary modalities. Periapical radiodensity was monitored on standardized digital periapical radiographs (Fona XDC unit with a Rinn XCP film holder for reproducible geometry; Digora software, version 2.0), expressed as grayscale values (0–255) at mesial, distal, and apical regions at baseline, 3, and 6 months (Fig. 4); values are reported as relative grayscale rather than direct bone density, as they are exposure-sensitive, semi-quantitative, and uncalibrated against a density phantom. Exact exposure settings and ROI geometry were not centrally logged, a limitation (see Limitations). To limit radiation exposure (ALARA principle), only a single postoperative CBCT was acquired, at the 6-month endpoint, for three-dimensional quantification of peri-implant radiodensity. Clinical evaluation (implant survival, infection, mobility, dehiscence) was performed weekly for 1 month and monthly thereafter.

**Fig. 5:**
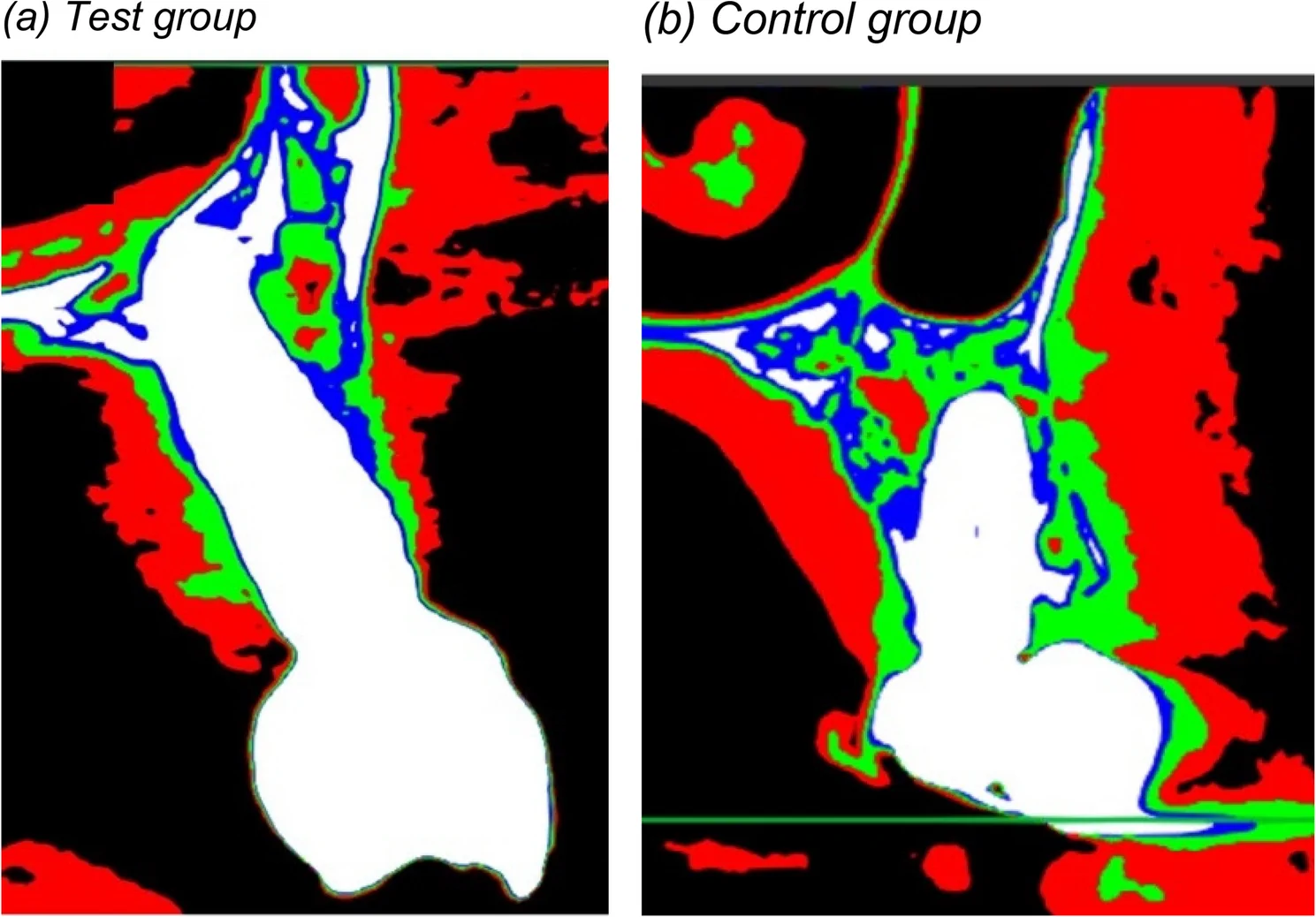
Six-month cone-beam CT radiodensity assessment (Atomica software; secondary, exploratory outcome). Color-coded peri-implant radiodensity maps showing the mean relative radiodensity (arbitrary gray-value units) for a representative test-group implant (**a**) and a representative control-group implant (**b**). Although the software interface displays the label "HU," these values represent uncalibrated scanner-specific gray values and should not be interpreted as true Hounsfield units. Note: The completed CONSORT 2010 checklist is provided as a separate supplementary file accompanying this submission

On the 6-month CBCT, peri-implant radiodensity was measured with Atomica software using its density-measurement tool. Three readings were obtained along the buccal aspect of each implant (jumping-gap area) at the apical, middle, and crestal levels, and the mean was recorded as CBCT-derived radiodensity (arbitrary gray-value units; Fig. 5). Because these values are not equivalent to true Hounsfield units and depend on scanner and acquisition parameters, they were interpreted as relative radiodensity. To improve reliability, measurements were performed independently by two blinded oral and maxillofacial radiologists (PhD), each obtaining two readings per measurement; intra- and inter-observer reliability were quantified by ICC. All scans were acquired on a single unit (SCANORA 3D; SOREDEX, PaloDEx Group Oy, Tuusula, Finland) with fixed exposure parameters (90 kVp, 4.0 mA, 20.0 s; voxel size 133 μm; field of view 14 × 16.5 cm) for all participants, and analyzed with Atomica software (version 5.0). Reconstruction and metal-artifact-reduction settings were not separately logged beyond this fixed protocol, a limitation (see Limitations); using identical acquisition parameters throughout was the principal step taken to minimize between-scan variability in beam-hardening and scatter artifacts. Porous β-TCP remains radiographically detectable for months after placement [9, 17], so radiodensity values in the test group may partly reflect residual graft material rather than newly formed bone (see Discussion). No density-calibration phantom was used, so all CBCT-derived measurements are reported consistently as relative, arbitrary gray-value radiodensity.

### Statistical analysis

Data were analyzed using SPSS (version 26.0); normality was assessed with the Shapiro-Wilk test, and data are expressed as mean ± SD. The patient served as the unit of analysis because randomization occurred at the patient level; averaging implant-level measurements within each patient was pre-specified, before data collection, to avoid pseudoreplication. Within-group changes over time were evaluated with repeated-measures ANOVA (Bonferroni-corrected post hoc), and between-group comparisons with the independent-samples t test (Welch’s correction for the 6-month CBCT comparison, where equality of variances could not be assumed). Because ISQ and periapical values were measured repeatedly within patients, we additionally fitted a two-way mixed-design ANOVA (group×time) for each outcome to test the interaction directly, with the change from baseline to 6 months as a robustness check. For the periapical outcome, given the small numerical baseline difference noted in Results, we additionally fitted an ANCOVA of the 6-month value on group, adjusting for baseline. Treatment-effect estimates are reported with 95% confidence intervals. A *p* value ≤ 0.05 was considered significant. The 6-month CBCT summary statistics were recalculated directly from the per-patient dataset during analysis preparation; this recalculation is used consistently throughout the Results, Table 5, and Discussion, superseding the previously reported values (see Limitations).

## Results

All analyses were performed at the patient level (eight patients per group). For patients who received more than one implant, the implant measurements were averaged to provide a single value per patient; accordingly, the reported statistics correspond to 16 patients rather than to the 39 implants placed, implant distribution by group is presented descriptively in Table 1.

### Demographic and baseline characteristics

Sample composition and baseline demographic characteristics are summarized descriptively in Table 1. Because this was a randomized, not matched, study, the non-significant between-group comparisons at baseline do not confirm baseline equivalence or comparability; they are reported descriptively only. Seven of eight patients in the control group (87.5%) and all eight patients in the test group (100%) were female, with no significant difference in sex distribution (*p* = 0.301). Mean age did not differ significantly between groups (control, 46 ± 5.6 years; test, 43 ± 4.8 years; *p* = 0.27).

### Jumping gap

The mean jumping gap did not differ significantly between groups (control, 2.94 ± 0.85 mm; test, 3.15 ± 0.94 mm; *p* = 0.372). In both groups the mean gap exceeded 2 mm, confirming that all included sites fulfilled the predefined inclusion criterion.

### Implant stability (ISQ)

Between-group comparison of ISQ revealed no significant differences at any time point: baseline (control, 55.00 ± 14.58; test, 50.88 ± 10.86; *p* = 0.53), 3 months (63.13 ± 9.63 versus 61.00 ± 7.25; *p* = 0.63), and 6 months (70.88 ± 9.67 versus 65.00 ± 8.88; *p* = 0.23). Within-group analysis demonstrated a significant overall increase in ISQ over time in both groups (control, *p* = 0.04; test, *p* = 0.02). Post hoc comparisons indicated that the significant improvement occurred primarily between baseline and 6 months, with no significant differences between baseline and 3 months or between 3 and 6 months in either group (Table 2).

**Table 2:** Implant stability quotient (ISQ) over time by group

Time point	Control (mean ± SD)	Test (mean ± SD)	Mean difference (95% CI)	*p*
Baseline	55.00 ± 14.58	50.88 ± 10.86	−4.1 (− 17.9, 9.7)	0.53
3 months	63.13 ± 9.63	61.00 ± 7.25	−2.1 (− 11.3, 7.0)	0.63
6 months	70.88 ± 9.67	65.00 ± 8.88	−5.9 (− 15.8, 4.1)	0.23

Mean difference is test minus control; negative values indicate higher ISQ in the control group. All between-group comparisons were non-significant, and all 95% confidence intervals included zero. Within-group ISQ increased significantly over time in both groups (control, *p* = 0.04; test, *p* = 0.02), mainly between baseline and 6 months

These within-group comparisons do not by themselves establish a between-group difference in the rate of change over time. The mixed-design ANOVA testing the group×time interaction for ISQ was not significant (F(2,28) = 0.72, *p* = 0.49), and the main effect of group was also not significant (F(1,14) = 0.69, *p* = 0.42), consistent with the between-group comparisons at each time point above. The independent-samples comparison of the change in ISQ from baseline to 6 months likewise showed no significant between-group difference (control + 15.9 ± 7.9; test + 14.1 ± 6.1; mean difference − 1.8, 95% CI − 9.4 to 5.9; t = − 0.50, df = 13.2, *p* = 0.63).

### Peri-implant radiodensity: periapical assessment

Between-group comparison of periapical grayscale value showed no significant differences at baseline (control, 123.13 ± 21.96; test, 105.13 ± 12.67; *p* = 0.06), 3 months (137.88 ± 17.42 versus 143.88 ± 11.85; *p* = 0.43), or 6 months (160.38 ± 15.51 versus 169.63 ± 13.37; *p* = 0.22). Within-group analysis demonstrated significant changes over time in both groups (*p* < 0.001); the test group reached significance at every interval, whereas the control group reached significance only between baseline and 6 months (Table 3). The mixed-design ANOVA testing the group×time interaction was significant (F(2,28) = 5.58, *p* = 0.009), and the change from baseline to 6 months was greater in the test group (control + 37.3 ± 24.4; test + 65.8 ± 24.2; mean difference 28.5, 95% CI 2.5 to 54.5; t = 2.35, df = 14.0, *p* = 0.034). Because the test group had a numerically lower baseline value (Table 3), we additionally fitted an ANCOVA of the 6-month value on group, adjusting for baseline; the adjusted group effect was not significant (mean difference 7.7 grayscale units, SE 8.6; t = 0.90, df = 13, *p* = 0.39), and baseline was not a significant covariate (*p* = 0.72). These findings are interpreted in the Discussion.

**Table 3:** Peri-implant radiodensity on periapical radiographs (grayscale values, 0–255) over time by group

Time point	Control (mean ± SD)	Test (mean ± SD)	Mean difference (95% CI)	*p*
Baseline	123.13 ± 21.96	105.13 ± 12.67	−18.0 (− 37.2, 1.2)	0.06
3 months	137.88 ± 17.42	143.88 ± 11.85	6.0 (− 10.0, 22.0)	0.43
6 months	160.38 ± 15.51	169.63 ± 13.37	9.3 (− 6.3, 24.8)	0.22

Mean difference is test minus control. All between-group comparisons were non-significant, and all 95% confidence intervals included zero. Within-group change was significant in both groups (*p* < 0.001); the test group reached significance across all intervals, whereas the control group reached significance only between baseline and 6 months

### Peri-implant radiodensity: 6-month CBCT

On the 6-month CBCT, mean relative peri-implant radiodensity, recalculated directly from the per-patient dataset, was higher in the test group (1224.0 ± 237.0) than in the control group (931.9 ± 100.4), a mean difference of 292.1 units (95% CI 87.7 to 496.5) favoring the test group (Tables 4, 5 and Fig. 5). This difference was statistically significant (Welch’s t = 3.21, df = 9.43, *p* = 0.010; Cohen’s d = 1.61). This outcome was not specified in the a priori sample-size calculation, which was powered only for ISQ (see Sample-size calculation); interpretation of this finding, including the possible contribution of residual graft material, is addressed in the Discussion.

**Table 4:** Group×time interaction and baseline-adjusted analyses for ISQ and periapical grayscale value

Outcome	Analysis	Statistic	df	*p* value
ISQ	Group×time interaction (mixed ANOVA)	F = 0.72	2, 28	0.49
ISQ	Main effect of group (mixed ANOVA)	F = 0.69	1, 14	0.42
ISQ	Change baseline→6mo, test vs. control	t = − 0.50	13.2	0.63
Periapical grayscale	Group×time interaction (mixed ANOVA)	F = 5.58	2, 28	0.009
Periapical grayscale	Change baseline→6mo, test vs. control	t = 2.35	14.0	0.034
Periapical grayscale	ANCOVA, group effect adjusted for baseline	t = 0.90	13	0.39

Mixed-design ANOVA: between-subjects factor group, within-subjects factor time (baseline, 3, 6 months); df given as (numerator, denominator). Change baseline→6mo compared between groups with an independent-samples t test (Welch). ANCOVA: 6-month periapical grayscale value regressed on group with baseline value as covariate; adjusted mean difference (test vs. control) 7.7 units (SE 8.6). Derived from the per-patient dataset supplied by the authors; see Statistical analysis and Results

**Table 5:** Relative peri-implant radiodensity measured on CBCT (arbitrary units) at 6 months by group (secondary, exploratory outcome)

Parameter	Control (*n* = 8)	Test (*n* = 8)	*p* value
Radiodensity, arbitrary gray-value units (mean ± SD)	931.9 ± 100.4	1224.0 ± 237.0	0.010

Values are relative peri-implant radiodensity in arbitrary units (not true Hounsfield units), recalculated directly from the per-patient dataset (see Statistical analysis note). Mean difference 292.1 units (95% CI 87.7 to 496.5) in favour of the test group; this outcome was not specified in the a priori sample-size calculation. Between-group comparison by independent-samples t test with Welch’s correction (t = 3.21, df = 9.43, *p* = 0.010; Cohen’s d = 1.61)

CBCT-derived radiodensity measurements demonstrated excellent reproducibility: the intra-observer ICC was 0.98 for the first radiologist and 0.96 for the second, and the inter-observer ICC was 0.92, indicating excellent intra- and interobserver reliability.

All 39 implants survived to the 6-month follow-up (implant-level survival, 39/39; 100%), and all 16 patients remained free of implant failure at the patient level (16/16; 100%). No adverse events, including signs of infection, implant mobility, or wound dehiscence, were observed in either group at any assessed level during the follow-up period.

## Discussion

This randomized controlled trial evaluated whether β-TCP grafting of peri-implant jumping gaps exceeding 2 mm improves implant stability and peri-implant radiodensity after immediate placement, an area in which recent systematic-review evidence remains inconclusive [18] Grafting did not significantly alter implant stability at any time point, including in a formal group×time interaction analysis. For the periapical outcome, the unadjusted rate of change differed significantly between groups, favoring the test group, but this difference was no longer significant after adjustment for a small numerical baseline difference and should therefore be interpreted as hypothesis-generating rather than a confirmed treatment effect. Standardized eligibility criteria and treatment protocols enhanced internal validity, and the strict inclusion and exclusion criteria minimized confounders such as systemic conditions, impaired healing, and inadequate oral hygiene, which are recognized contributors to compromised osseointegration and implant failure [1, 2].

A key feature of the study is its focus on jumping gaps greater than 2 mm, a threshold widely regarded as clinically critical. Defects beyond this dimension have been suggested to demonstrate limited predictability for spontaneous bone regeneration when reliant on blood-clot stabilization alone [7], a view supported by Botticelli et al., who reported that increasing gap width may adversely influence bone-healing dynamics and predispose to marginal bone loss [6]. Restricting the sample to defects exceeding 2 mm therefore provided a framework in which to assess grafting in scenarios where intervention is most likely to be justified.

The study was supported by an a priori sample-size calculation for the primary ISQ outcome [19], and computerized allocation with concealed, sequentially numbered envelopes reduced selection bias. Atraumatic extraction was used consistently to preserve the buccal plate, which is highly susceptible to post-extraction resorption [20], and implant-bed preparation followed a prosthetically driven, slightly palatal approach with apical extension to engage native bone and optimize primary stability, consistent with contemporary immediate-placement protocols [21].

No significant between-group difference in implant stability was detected at any time point, consistent with the broader principle that primary stability is strongly influenced by surgical execution and implant positioning [22]. The increase in stability over time within both groups reflects the expected biological progression from primary mechanical stability to secondary, biologically mediated stability as osseointegration proceeds [23]. Given the small sample size and wide confidence intervals, adequately powered studies are needed before firm conclusions can be drawn about the specific contribution of gap grafting to stability.

Radiographically, the mixed-design ANOVA and the change-from-baseline comparison both indicated a significantly faster increase in periapical grayscale value in the test group (group×time interaction, F(2,28) = 5.58, *p* = 0.009; change-from-baseline mean difference 28.5 units, 95% CI 2.5 to 54.5, *p* = 0.034), but this was no longer significant after adjusting for the numerically lower baseline value in the test group (ANCOVA adjusted difference 7.7 units, *p* = 0.39). This pattern suggests that a substantial part of the apparent effect may reflect the small numerical baseline difference or random variation rather than a true treatment effect, and should be interpreted cautiously given the small sample.

Peri-implant radiodensity was monitored at intervals with standardized periapical radiographs, while a single CBCT was reserved for the 6-month endpoint to limit radiation exposure in accordance with the ALARA principle. On this CBCT, the grafted group showed significantly higher radiodensity than the control group (mean difference 292.1 arbitrary units; 95% CI 87.7 to 496.5; *p* = 0.010; Cohen’s d = 1.61), recalculated directly from the per-patient dataset. This comparison was not specified in the a priori sample-size calculation, which powered only the ISQ outcome, so despite reaching significance it should be interpreted as a secondary, exploratory finding. Because no density-calibration phantom was used and CBCT gray values reflect the combined attenuation of bone and any residual graft material rather than bone alone, they cannot independently confirm new bone formation or reliably distinguish newly formed bone from residual β-TCP; porous β-TCP is resorbed and replaced by host bone over approximately 6–12 months and remains radiographically and histologically detectable well beyond 3 months [9, 17, 24], so some portion of the higher test-group radiodensity may reflect residual graft material rather than new bone. Any true biological effect would be consistent with the osteoconductive properties of β-TCP, which provides a scaffold supporting cellular migration, angiogenesis, and osteoblastic activity [9]; in wide gaps, such a scaffold could in principle enhance early defect fill, but the present data do not establish this, and the finding remains hypothesis-generating pending confirmation in adequately powered prospective trials.

These findings are consistent with previous investigations of β-TCP in immediate-implant scenarios. In a randomized clinical trial, Daif reported no significant difference in implant survival between β-TCP-grafted and non-grafted sites at 6 months post-loading [11]. They also align with a 2025 systematic review and meta-analysis of randomized trials of flapless immediate implants, which found that grafting the peri-implant gap may enhance alveolar bone preservation, particularly at the deeper aspect of the buccal wall, while emphasizing that further high-quality trials are required [18]. These observations are in keeping with the experimental work of Botticelli et al., who showed that gap dimension influences peri-implant healing dynamics [6], and with Araújo and Lindhe, who documented the pronounced dimensional changes of the buccal wall after extraction [20]. The present study adds to this evidence by focusing specifically on gaps exceeding 2 mm. Collectively, the results demonstrated no statistically significant between-group difference in implant stability or in baseline-adjusted periapical grayscale values; however, 6-month CBCT-derived radiodensity was significantly higher in the β-TCP group. A methodological distinction between the two radiographic modalities may help explain this divergence: the periapical grayscale value was sampled at mesial, distal, and apical regions of the implant, none of which directly overlie the buccal jumping gap, whereas the CBCT measurement was obtained specifically along the buccal aspect of the implant at the jumping-gap site itself, where the injected β-TCP was placed. Because the CBCT measurements were obtained directly from the buccal jumping-gap region, they more directly sampled radiodensity at the grafted defect site than did the periapical measurements, which were taken mesial and distal to the implant. Given that the CBCT outcome was not prospectively powered and may partly reflect residual graft material, this finding should nonetheless be interpreted cautiously. Because the trial was not designed or powered as an equivalence or non-inferiority study, larger, equivalence-designed trials are needed before conclusions about the necessity of grafting can be drawn.

### Limitations and future directions

Several limitations should be considered. First, the sample was relatively small (eight patients per group) and follow-up was restricted to early healing; the radiographic outcomes lacked a formal a priori power target, so both should be confirmed in a purpose-designed study. Second, the trial was registered retrospectively rather than prospectively; prospective registration should be prioritized in future trials. Third, radiodensity was assessed radiographically rather than volumetrically: periapical grayscale values are exposure-sensitive and semi-quantitative, and the CBCT-derived relative radiodensity values, although reproducible under this study’s fixed acquisition protocol, are not calibrated to true Hounsfield units and do not capture bone volume or microarchitecture directly. Fourth, soft-tissue and histological outcomes were not evaluated, and a single alloplastic material was studied at a single center in maxillary anterior and premolar sites only, which limits generalizability to other sites and bone densities. Fifth, the implant diameter/length reported in Supplementary Table S1 corresponds to a single index implant per patient rather than every implant placed. Sixth, the 6-month CBCT summary statistics initially calculated for this report were subsequently corrected on recalculation directly from the per-patient dataset; the values throughout this manuscript and in Table 5 reflect the corrected figures, and no multiplicity correction was applied across the ISQ, periapical, and CBCT outcomes, which is itself a limitation. Seventh, the group×time and baseline-adjusted analyses used a mixed-design ANOVA and ANCOVA rather than a full linear mixed-effects model; given the small sample the two approaches are expected to give similar conclusions, but a mixed-effects model remains the more flexible and preferred approach.

Future research should incorporate larger, prospectively registered cohorts with longer follow-up; combine calibrated three-dimensional and volumetric imaging with histological and soft-tissue evaluation; and directly compare β-TCP with xenograft and allograft materials to establish more definitive clinical guidelines.

## Conclusions

In this randomized trial of immediate implants placed in sites with a jumping gap exceeding 2 mm, β-TCP grafting did not significantly affect implant stability (ISQ) at any time point, including in a formal group×time interaction analysis. Periapical grayscale value increased faster in the test group on unadjusted analysis, but this difference was no longer significant after adjustment for a small numerical baseline difference. The test group showed significantly higher 6-month CBCT-derived peri-implant radiodensity than the control group (mean difference 292.1 arbitrary units, 95% CI 87.7 to 496.5; *p* = 0.010). Because CBCT radiodensity cannot distinguish newly formed bone from residual graft material and this outcome was not prospectively powered, and because the trial was not designed to test equivalence, these findings—particularly the CBCT and periapical results—require confirmation in larger, adequately powered trials before conclusions about the necessity of grafting in jumping gaps exceeding 2 mm can be drawn.

## Supplementary Information

Supplementary Material 1.

Supplementary Material 2.

## Data Availability

The data generated and analyzed during this study are available from the corresponding author on reasonable request.

## Abbreviations

β-TCP: Beta-tricalcium phosphate
ISQ: Implant stability quotient
CBCT: Cone-beam computed tomography
HU: Hounsfield units (note: CBCT-derived measurements in this study are relative, arbitrary gray-value radiodensity units and are not calibrated true Hounsfield units)
RFA: Resonance frequency analysis
ANOVA: Analysis of variance
ICC: Intraclass correlation coefficient
SD: Standard deviation
CI: Confidence interval
ALARA: As low as reasonably achievable
