# Supplementary material for: Effect of β-tricalcium phosphate grafting on implant stability and peri-implant radiodensity following immediate implant placement in sites with jumping gaps exceeding 2 mm: a randomized controlled trial
Source: BMC Oral Health. 2026 Sep 21;26:1780. doi: 10.1186/s12903-026-09810-5 (PMC13591874; doi:10.1186/s12903-026-09810-5)
Supplement: Supplementary file 1 [file 12903_2026_9810_MOESM1_ESM.pdf]

CONSORT 2010 Checklist

Effect of β-Tricalcium Phosphate Grafting on Implant Stability and Peri-implant Radiodensity Following Immediate Implant Placement in Sites with Jumping Gaps Exceeding 2 mm: A Randomized Controlled Trial

Title and abstract

Item	Checklist item	Reported in (manuscript)
1a	Identification as a randomized trial in the title	Title, p. 1
1b	Structured summary of trial design, methods, results, and conclusions	Abstract, p. 2 (condensed to within the journal's 350-word limit)

Introduction

Item	Checklist item	Reported in (manuscript)
2a	Scientific background and rationale	Background, p. 3
2b	Specific objectives or hypotheses	Background (null hypothesis stated), p. 3–4

Methods

Item	Checklist item	Reported in (manuscript)
3a	Description of trial design, including allocation ratio	Study design and ethical approval, p. 4; Randomization and blinding (1:1 ratio), p. 6
3b	Important changes to methods after trial commencement	Not applicable
4a	Eligibility criteria for participants	Participants, p. 4; eligibility timing clarified in Randomization and blinding, p. 6
4b	Settings and locations where data were collected	Study design and ethical approval, p. 4
5	Interventions for each group with sufficient detail	Surgical procedure, p. 6

6a	Completely defined pre-specified primary and secondary outcomes	Outcome measures, p. 7 (primary comparison: ISQ at 6 months, defined explicitly; secondary: periapical grayscale value and 6-month CBCT relative radiodensity)
6b	Any changes to trial outcomes after commencement	Not applicable
7a	How sample size was determined	Sample-size calculation, p. 4 (includes the formula, α and power, literature-based justification of the clinically relevant difference, dropout allowance, patient-level unit-of-analysis statement, and radiographic-outcome power caveat; the standalone sample-size table was removed per reviewer request and its content folded into the Methods text)
7b	Interim analyses and stopping guidelines	Not applicable
8a	Method used to generate the random allocation sequence	Randomization and blinding, p. 6
8b	Type of randomization; details of any restriction	Randomization and blinding (1:1 ratio, no stratification), p. 6
9	Mechanism used to implement allocation (concealment)	Randomization and blinding (sequentially numbered, opaque, sealed envelopes), p. 6
10	Who generated the sequence, enrolled, and assigned participants	Randomization and blinding, p. 6
11a	Blinding: if done, who was blinded and how	Randomization and blinding, p. 6; Outcome measures (blinded radiographic assessors), p. 7
11b	Similarity of interventions	Surgical procedure, p. 6 (surgeon and patient not blinded to allocation; outcome assessors blinded)

12a	Statistical methods for primary and secondary outcomes	Statistical analysis, p. 8
12b	Methods for additional analyses	Statistical analysis, p. 8 (mixed-design ANOVA testing the group×time interaction for ISQ and periapical grayscale value; ANCOVA for periapical grayscale value adjusting for the small numerical baseline difference)

Results

Item	Checklist item	Reported in (manuscript)
13a	Numbers randomized, treated, and analyzed per group	Results, p. 9; CONSORT flow diagram, Figure 1 (main manuscript)
13b	Losses and exclusions after randomization	CONSORT flow diagram, Figure 1; none occurred (0 exclusions, 0 losses to follow-up)
14a	Dates defining periods of recruitment and follow-up	Study design and ethical approval, p. 4
14b	Why the trial ended or stopped	Not applicable (completed as planned)
15	Baseline demographic and clinical characteristics	Table 1, p. 19 (merged sample-composition and baseline-characteristics table, per reviewer request)
16	Numbers analyzed in each group	Results, p. 9; Table 1, p. 19
17a	Outcomes, effect size, and precision (95% CI)	Results, p. 9–11; Tables 2–4, p. 20–21; Table 5 (group×time interaction and ANCOVA estimates), p. 22
17b	For binary outcomes, absolute and relative effect sizes	Not applicable

18	Results of any other analyses performed	Results (inter-/intra-observer reliability, ICC), p. 11; group×time interaction and baseline-adjusted (ANCOVA) analyses, Results p. 9–11 and Table 5, p. 22
19	All important harms or unintended effects	Results (implant-level survival 39/39; patient-level freedom from implant failure 16/16; no adverse events), p. 11

Discussion

Item	Checklist item	Reported in (manuscript)
20	Trial limitations, sources of potential bias and imprecision	Discussion (Limitations), p. 14
21	Generalizability (external validity)	Discussion (Limitations), p. 14
22	Interpretation consistent with results and balance of evidence	Discussion, p. 12–15 (interpretation of the 6-month CBCT radiodensity finding revised for consistency with the significant between-group result, p = 0.010)

Other information

Item	Checklist item	Reported in (manuscript)
23	Registration number and name of trial registry	Declarations, p. 16; ClinicalTrials.gov NCT07709676 (retrospectively registered; acknowledged as a limitation, p. 14)
24	Where the full trial protocol can be accessed	Available from corresponding author on request
25	Sources of funding and other support	Declarations (Funding), p. 16

Note: The CONSORT 2010 flow diagram is presented as Figure 1 in the main manuscript, consistent with its position as the first figure cited in the text. This completed checklist is provided as a separate supplementary file, per editorial instruction.
