# Supplementary material for: Effect of β-tricalcium phosphate grafting on implant stability and peri-implant radiodensity following immediate implant placement in sites with jumping gaps exceeding 2 mm: a randomized controlled trial
Source: BMC Oral Health. 2026 Sep 21;26:1780. doi: 10.1186/s12903-026-09810-5 (PMC13591874; doi:10.1186/s12903-026-09810-5)
Supplement: Supplementary file 2 [file 12903_2026_9810_MOESM2_ESM.docx]

# **Supplementary Data**

**Effect of β-Tricalcium Phosphate Grafting on Implant Stability and Peri-implant Radiodensity Following Immediate Implant Placement in Sites with Jumping Gaps Exceeding 2 mm: A Randomized Controlled Trial**

**Supplementary Table S1.** Implant diameter and length (mm) for the index implant in each patient, by group.

| **Group** | **Patient** | **Diameter × length (mm)** |
| --- | --- | --- |
| Control | Patient 1 | 3.7 × 14 |
| Control | Patient 2 | 4.1 × 14 |
| Control | Patient 3 | 3.7 × 12 |
| Control | Patient 4 | 4.1 × 14 |
| Control | Patient 8 | 4.1 × 10 |
| Control | Patient 10 | 3.3 × 12 |
| Control | Patient 11 | 4.1 × 14 |
| Control | Patient 16 | 3.3 × 14 |
| Test | Patient 5 | 4.1 × 12 |
| Test | Patient 6 | 4.1 × 14 |
| Test | Patient 7 | 3.3 × 10 |
| Test | Patient 9 | 3.3 × 12 |
| Test | Patient 12 | 3.7 × 14 |
| Test | Patient 13 | 3.3 × 14 |
| Test | Patient 14 | 4.1 × 10 |
| Test | Patient 15 | 3.3 × 14 |

*One index implant per patient is reported; patients with multiple implants received more than one, as summarized in Table 1.*
